# Genetic Modifiers of Hemoglobin Expression from a Clinical Perspective in Hemoglobinopathy Patients with Beta Thalassemia and Sickle Cell Disease

**DOI:** 10.3390/ijms252211886

**Published:** 2024-11-05

**Authors:** Michael D. Diamantidis, Georgia Ikonomou, Ioanna Argyrakouli, Despoina Pantelidou, Sophia Delicou

**Affiliations:** 1 Department of Hematology, Thalassemia and Sickle Cell Disease Unit, General Hospital of Larissa, 41221 Larissa, Greece; ioanna-arg@hotmail.com; 2 Thalassemia and Sickle Cell Disease Prevention Unit, General Hospital of Larissa, 41221 Larissa, Greece; georgia.ikonomou@gmail.com; 3 Thalassemia and Sickle Cell Disease Unit, AHEPA University General Hospital, 41221 Thessaloniki, Greece; dpantelidou@yahoo.gr; 4 Center of Expertise in Hemoglobinopathies and Their Complications, Thalassemia and Sickle Cell Disease Unit, Hippokration General Hospital, 41221 Athens, Greece; sophiadelicou@hippocratio.gr

**Keywords:** hemoglobin expression, genetic modifiers, fetal (α_2_γ_2_) hemoglobin (HbF), adult (α_2_β_2_) hemoglobin (HbA), HbA_2_ (α_2_δ_2_), hereditary persistence of fetal hemoglobin (HPFH), γ-globin gene expression, β-thalassemia, sickle cell disease (SCD), hemoglobinopathies, hydroxyurea (HU), DNA methylation, histone modification, gene editing, gene therapy

## Abstract

Hemoglobinopathies, namely β-thalassemia and sickle cell disease (SCD), are hereditary diseases, characterized by molecular genetic aberrations in the beta chains of hemoglobin. These defects affect the normal production of hemoglobin with severe anemia due to less or no amount of beta globins in patients with β-thalassemia (quantitative disorder), while SCD is a serious disease in which a mutated form of hemoglobin distorts the red blood cells into a crescent shape at low oxygen levels (qualitative disorder). Despite the revolutionary progress in recent years with the approval of gene therapy and gene editing for specific patients, there is an unmet need for highlighting the mechanisms influencing hemoglobin production and for the development of novel drugs and targeted therapies. The identification of the transcription factors and other genetic modifiers of hemoglobin expression is of utmost importance for discovering novel therapeutic approaches for patients with hemoglobinopathies. The aim of this review is to describe these complex molecular mechanisms and pathways affecting hemoglobin expression and to highlight the relevant investigational approaches or pharmaceutical interventions focusing on restoring the hemoglobin normal function by linking the molecular background of the disease with the clinical perspective. All the associated drugs increasing the hemoglobin expression in patients with hemoglobinopathies, along with gene therapy and gene editing, are also discussed.

## 1. Introduction

The term ‘thalassemia’ describes a group of hereditary hematological disorders characterized by a partial or total deficiency in the synthesis of the β-globin chain of hemoglobin. This deficiency leads to insufficient erythropoiesis and chronic anemia. Despite the significant advantages that conventional therapies, including iron chelation therapy and regular blood transfusions, provide, they are unable to resolve the genetic defect that is the primary cause of the condition [1,2,3].

Human hemoglobin is a tetramer protein of 2 α (alpha) and 2 β (beta) globular subunits, conjugated with the heme molecule. Studying hemoglobin and the relevant genes affecting its expression has been a major field of molecular hematological research for many years. Actually, globin genes were the first to be cloned, whereas hemoglobin was the first complex protein to have its composition totally defined [4,5]. The α-globin gene locus is found on the short arm of chromosome 16 in the genomic area p13.3, while the β-globin gene cluster is located on the short arm of chromosome 11 in the genomic area p15.5 [6,7].

A shift from γ-globin to β-globin gene expression characterizes the switch from fetal (α_2_γ_2_) hemoglobin (HbF) to adult (α_2_β_2_) hemoglobin (HbA) production. This normal ontogenic procedure starts taking place shortly after birth. Consequently, by the 6th month of age of the toddler, the major hemoglobin is HbA [8,9,10]. In normal adult life, HbF is below 2%, HbA is the major hemoglobin (>95%), whereas HbA_2_ (α_2_δ_2_) is less than 3.5% [10]. Nevertheless, the aforementioned hemoglobin switch is not irreversible. All adults retain the ability to produce levels of residual HbF at different degrees, forming namely the hereditary persistence of fetal hemoglobin (HPFH) [8,10].

Deletions or point mutations in the proximal promoter of γ-globin genes (*HBG1*, *HBG2*) have been linked to high γ-globin gene expression and HPFH [11]. Clinical, genetic, and biochemical findings led to the conclusion of a possible beneficial role for HbF (and increased γ-globin gene expression) in patients with β-thalassemia and sickle cell disease (SCD). Moreover, partially reversing the γ- to β-globin switch is the hallmark of designing proper therapeutic strategies for these hemoglobinopathies [4,9]. Nevertheless, the exact mechanisms regarding the switch from fetal to adult hemoglobin are under investigation. This switch depends on and is regulated by several complex genetic modifiers.

In general, hemoglobin expression is subject to control by various molecules or mechanisms, which modify its function [4,11]. The study of these genetic modifiers of hemoglobin expression has been a major research field in hemoglobinopathies. The aim of the present review is to shed light on these complex mechanisms and to highlight ways of targeting these pathways in order to perform pharmaceutical interventions, especially in the era of gene therapy and gene editing. Recent advances in gene therapy and gene editing have created fascinating opportunities for developing potentially therapeutic methods for correcting the genetic abnormalities responsible for theoretically all types of hemoglobinopathies. However, due to the very high cost and the limited number of patients, gene therapy and gene editing are addressed too. There is a vast unmet need for the discovery of novel targeted drugs for all patients with hemoglobinopathies and not for a small minority.

## 2. Hemoglobin Expression

**Figure 1:**
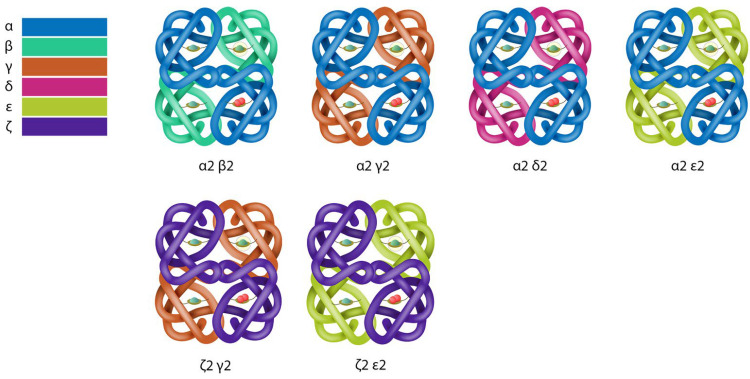
The various types and structure of human hemoglobin (see text).

The human hemoglobin molecule comprises four globin chains, two α-like chains and two β-like chains. There are two α-globin genes, ζ- and α- (*HBA1*, *HBA2*), and they are referred to as α-like genes. The human β-globin gene cluster consists of four genes arranged in the same order of expression, as it is during development, ε-, γ- (*HBG1*, *HBG2*), δ-, and β-globin genes, and they are referred to as β-like genes [12]. During the fetus’s first three months, only one α-like gene (ζ) and one β-like gene (ε) are activated, which produce ζ and ε chains, forming hemoglobin Gower-1 (ζ_2_ε_2_). Soon after that, α and γ chain synthesis begins, leading to the production of Hb Gower-2 (α_2_ε_2_) and Hb Portland (ζ_2_γ_2_). Next, in the development of the fetus, ζ and ε synthesis ceases and α and γ chains are produced, which join to form HbF (α_2_γ_2_), also known as fetal hemoglobin. By 17 weeks, only 1% of the cells continues to express ζ-globin [13]. The various types of human hemoglobin are shown in Figure 1.

During the first half year of life of the toddler, γ chain synthesis steadily decreases and is substituted by β chain synthesis, so that adult hemoglobin HbA (α_2_β_2_) is formed. The remaining globin gene, δ, becomes activated at birth, producing δ chains at low levels, which join with α chains to manufacture the second adult hemoglobin, HbA_2_ (α_2_δ_2_). Normal adults produce HbA (95%), HbA_2_ (<3.5%), and HbF (<1%–2%). Individuals with mutations in the γ-globin promoter or deletions of the β-globin gene can express higher levels of HbF, and this is called HPFH [14].

The regulation of gene expression is performed in every developmental stage, so that β-globin chains are equal to α-globin chains. This is the result of fine transcriptional, post-transcriptional, and post-translational control by which the transcriptional excess of the two α-genes achieves the effect of the single β-globin gene [15].

Furthermore, the expression of α-globin-like genes starts with the α-globin regulatory elements that lie upstream of the gene, where transcription factors restricted to the erythroid lineage bind [16]. This binding is essential for the induction of α-globin gene expression. The complex of GATA1, SCL, LDB1, E2A, LMO2, and other factors, such as NF-E2, recruit polymerase II to the α-gene locus, and then it facilitates the relocation of polymerase II to the promoter [17]. This last phase in the initiation of α-gene transcription starts with a variety of chromatin alterations. The transcription starts and transcripts are accumulated, once the polymerase II is positioned on the promoter. Active transcription takes place primarily in the basophilic erythroblast and in the polychromatophilic erythroblast [18].

**Figure 2:**
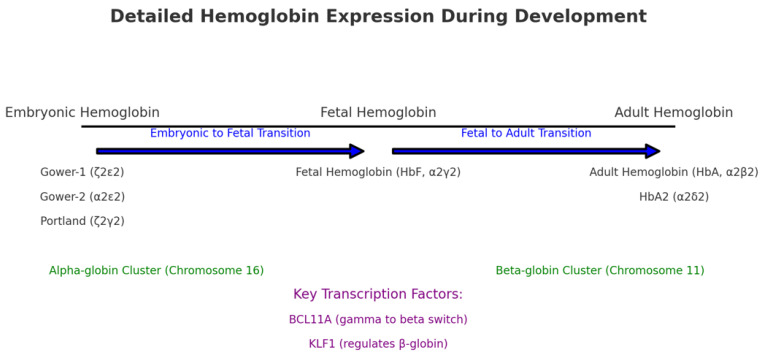
Main modifications of hemoglobin expression from embryonic to fetal and from fetal to adult hemoglobin.

The process of β-globin switch is a complex procedure of both *cis*-acting elements and *trans*-acting regulatory proteins. Via the looping of the globin locus, the Locus Control Region (LCR) is engaged to each globin gene in a sequence depending on restricted transcriptional factors, based on the phase of development [19]. This DNA looping facilitates the activation of particular genes through the activity of the promoters of distal enhancers [20], and thus complicated genetic and epigenetic molecular systems might affect gene expression [21,22]. For example, at embryonic life, the LCR is recruited to the γ-globin genes. Throughout adult life, repressors connect to the γ-globin promoter, inhibiting the attachment of activators and allowing BCL11A and KLF1 to attach the LCR to the β-globin gene [19]. Importantly, DNA methylation is an epigenetic event, silencing γ-globin genes in adult erythroid cells [23]. Post-translational modifications of histones, including histone acetylation and methylation, are also implicated in the alteration in the transcription of the γ-globin gene [24]. Histone acetylation is molecularly linked to the open conformation of chromatin, thereby leading to elevated gene transcription. On the other hand, histone methylation can either inhibit or promote gene transcription, according to the position and the number of methylated amino acid residues [25]. In addition, miRNAs (small non-coding nucleotides) are involved in the post-transcriptional regulation of the transcription factors implicated in γ-globin gene expression [26]. Figure 2 describes the main modifications of hemoglobin expression from embryonic to fetal and from fetal to adult hemoglobin.

The clinical data show that the severity of β-hemoglobinopathies can be improved through elevated HbF production [27,28]. The elevated expression of the γ-globin gene is an established disease modifier [29].

## 3. Genetic Modifiers of Hemoglobin Expression

**Figure 3:**
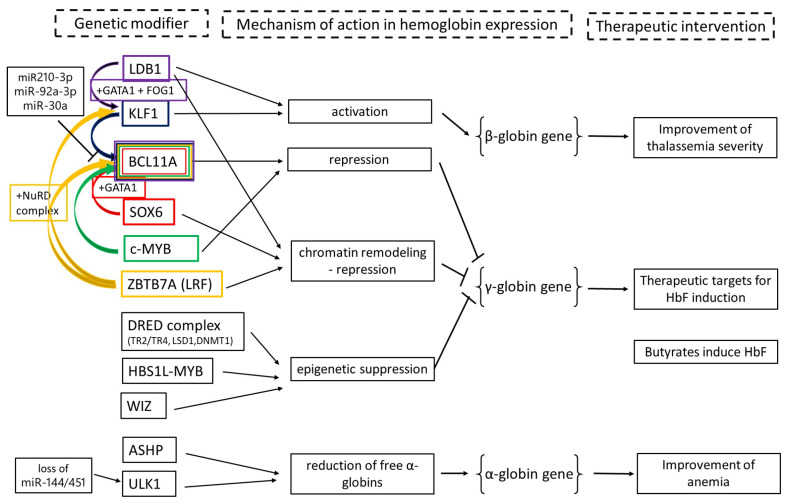
Genetic modifiers of hemoglobin expression and clinical implications in hemoglobinopathies.

The following genetic modifiers of hemoglobin expression are described in this section. Table 1 provides an overall summary of these modifiers and their relevant mechanisms, the possible mechanisms of therapeutic intervention, and the possible role of the described genetic modifiers in affecting hemoglobin expression. Figure 3 provides a graphical description of Table 1 for a better understanding of these important concepts.

The Erythroid Krüppel-like transcription factor (EKLF/KLF1) is located on chromosome 19p13.12–19p13.13. It is a transcription factor expressed in hematopoietic organs from a very early stage of embryogenesis and during the organism’s lifespan [30]. It plays a vital role in erythropoiesis [31,32] and is present in both primitive and definitive erythroid populations [33]. KLF1 activates genes that encode globins, heme synthesis enzymes, and other proteins related, directly or not, to erythropoiesis [34] and modulates the expression of other transcription factors, which cooperate to control gene expression in erythroid cells [35,36,37,38]. KLF1 variants affect red blood cell phenotypes in many different ways: they reduce the expression of many antigens, like Lutheran, Kell, Duffy, and Kidd [39,40]; they dysregulate globin expression while switching from fetal globin to adult β-globin [41]; and they reduce pyruvate kinase (PK) [42]. Furthermore, KLF1 variations correlate with variations in globin production, affecting the severity of thalassemia. KLF1 is also involved in globin switching by activating BCL11A or in the direct interaction with the β-promoter of the β-globin gene [43,44,45]. KLF1 recruits the histone chaperone HIRA, a fundamental process for β-globin gene expression [38]. Moreover, it is well known that a higher expression of HbF benefits adult β-thalassemia patients [46]. KLF1 via knockout mouse models is an established key player from a hemoglobinopathy clinical perspective [47] and a candidate participant in gene therapy for β-hemoglobinopathies [48].

**Table 1:** Genetic modifiers and relevant mechanisms, possible mechanisms of therapeutic interventions, and their possible role in hemoglobin expression. HbF: fetal hemoglobin, HPFH: hereditary persistence of fetal hemoglobin, and Ref: reference.

Genetic Modifier and Mechanism of Action—References	Mechanism of Therapeutic Intervention	Possible Role in Hemoglobin Expression
Promoter of γ-globin gene (ref. [4])	Deletion of 13 bp in the promoter causes a naturally occurring HPFH-associated mutation	Increased HbF expression
BCL11A–ZBTB7A (LRF) acts as A γ-globin gene repressor (refs. [49,50,51,52,53,54,55,56])	Disruption of the BCL11A enhancer by CRISPR-Cas9 genome editing	Inhibition of BCL11A–ZBTB7A increases γ-globin gene expression and HbF
SOX6 synergizes with BCL11A and GATA1 in the β-globin gene locus conducting the silencing of γ-globin transcription (refs. [57,58,59,60])	Knockdown studies confirm increased γ-globin production	SOX6 silencing for reactivating γ-globin and elevating HbF levels
DRED is an epigenetic complex leading to γ-globin promoter suppression (refs. [61,62,63])	Targeting DRED restores γ-globin promoter activation	The DRED complex is considered a target for agents to treat β-hemoglobinopathies
HBS1L-MYB gene polymorphisms associated with elevated HbF (refs. [64,65,66,67])	Target gene for hemoglobinopathy therapy	HbF induction
c-MYB directly activates γ-globin repressor genes (refs. [68,69,70,71,72,73,74,75,76,77])	Knockdown of the c-MYB gene increases HbF in human erythroid progenitors	Inhibition of the c-MYB gene increases γ-globin gene expression and HbF
DNA methyltransferases, histone deacetylases 1/2, and LSD1 act as epigenetic silencers of γ-globin and HbF expression (refs. [78,79,80,81])	The corresponding inhibitors in mice and in humans restore γ-globin production	Increased HbF expression after pharmacological inhibition
Butyrates act as HDACis and enhance γ-globin synthesis (refs. [82,83,84,85,86,87,88,89,90,91,92,93,94,95,96,97,98,99,100])	Activation of butyrates as HbF inducers	Induction of HbF production in patients with thalassemia intermedia
WIZ transcription factor acts as an HbF repressor (ref. [101])	Pharmacological WIZ degradation by dWIZ-1 and dWIZ-2 in humanized mice and cynomolgus monkeys	Robust induction of HbF by dWIZ-1 and dWIZ-2, the molecular glue degraders of WIZ
miR-210-3p miR-92a-3p miR-30a targeting BCL11A mRNA (refs. [102,103,104,105,106,107,108,109,110,111,112])	Inhibition of BCL11A	Upregulation of γ-globin expression
AHSP gene (α-globin genetic modifier) (refs. [113,114,115])	Increased expression of AHSP	Reduction in the toxic excess of free α-globins in β-thalassemia and SCD
ULK1 induces autophagy in erythroid cells (refs. [113,114,115])	Loss of miR-144/451 stimulates the ULK1-mediated autophagy of free α-globins	Reduction in free α-globins in β-thalassemia and SCD; improvement of anemia

B-cell lymphoma/leukemia 11A (BCL11A) is also named the C2H2-type zinc finger protein and is located on chromosome 2p16.1. It is a DNA-binding protein and a major transcription repressor of γ-globin expression during adult erythropoiesis. BCL11A, found within multiprotein transcription complexes, does not act alone, but co-operates with other transcription repressors, such as GATA1, TAL1, and the nucleosome remodeling and deacetylase (NuRD) complex, which is a chromatin regulator [4,21,49,50,51,52,53]. All these molecules form a network, inhibiting γ-globin expression and HbF in adults [52,53]. However, in the fetus, BCL11A expression is suppressed in order to maintain elevated amounts of HbF. In this regard, the inhibitor HIC2 connects to BCL11A enhancers to decrease the chromatin availability and binding of the transcription factor GATA1 [54]. Furthermore, other activators increase the expression of γ-globin genes in the fetal stage [52,55]. Interestingly, BCL11A haploinsufficiency has been associated with HPFH [56]. Gene editing aimed at addressing BCL11A might improve γ-globin expression, possibly leading to elevated HbF levels in patients with hemoglobinopathies and a better clinical outcome.

The SRY-box transcription factor-6 (SOX6) belongs to the SOX family, binds easily to DNA, and helps chromatin remodeling [57]. It is located on chromosome 11p15.2, the same chromosome in which the β-globin gene cluster is found. It acts in synergy with BCL11A and GATA1 in the β-globin gene cluster and inhibits γ-globin transcription [57]. Polymorphisms (SNPs) of the SOX6 gene do not alter HbF levels [58], but knockdown studies confirmed increased γ-globin production [59]. Therefore, SOX6 inhibition could serve as an important therapeutic target to increase γ-globin and elevate HbF levels [60].

The Direct Repeat Erythroid Definitive (DRED) complex is an epigenetic compound [61] consisting of orphan nuclear receptors TR2/TR4 [62], lysine specific demethylase 1 (LSD1, chromosome 1p36.12), and deoxynucleic acid methyltransferase 1 (DNMT1). It binds with increased molecular affinity to the direct repeat sites of ε- and γ-globin promoters, causing their inhibition by interacting with corepressors, such as NuRD and CoREST [22]. Because of this, the DRED complex is considered a target for agents to treat β-hemoglobinopathies [63].

The transcription factor zinc finger and BTB domain containing 7A (ZBTB7A), also known as the leukemia/lymphoma-related factor (LRF), is located on chromosome 19p13.3 in proximity with EKLF/KLF1 and WIZ transcription factors. This factor interacts with BCL11A, KLF1, and with components of the NuRD complex, repressing HbF indirectly and causing γ-globin silencing [116,117]. All these factors show chromatin-modifying properties and influence the epigenetic state of the globin cluster [4].

The variant HBS1-like translational GTPase-Myeloblastosis (HBS1L-MYB), located on chromosome 6q23.3, is an intergenic region linked to increased HbF levels and modifications of several clinically significant human erythroid characteristics. Various HBS1L-MYB intergenic variants influence regulatory molecular loci, occupied by key erythroid transcription factors [64]. Single Nucleotide Polymorphisms (SNPs) of the HBS1L-MYB gene are associated with elevated HbF levels in patients with β-major and thalassemia intermedia (TI) [65]. Even though the mechanism of this association is not clear yet, the two genes (HBS1L and MYB) are candidate target genes for hemoglobinopathy therapy [66,67].

c-MYB is an important regulator of erythropoiesis [64,68]. c-MYB is the cellular counterpart of the MYB transcription factor and is essential for survival. It is highly expressed in hematopoietic cells and is required for erythroid maturation [69]. It plays a fundamental role in controlling erythroid proliferation/differentiation balance [70] and sustains proliferation. Interestingly, low c-MYB levels favor accelerated differentiation [71]. RNA interference (RNAi) and gene knockout studies provided evidence that c-MYB is essential for hematopoiesis [72]. MYB is a relevant gene in the HBS1L-MYB region modifying erythroid characteristics and HbF levels. There are indications that the increased HbF levels are mediated through the down-modulation of MYB [73]. On the other hand, the forced expression of c-MYB significantly inhibits γ-globin expression [74]. The mechanism of how c-MYB controls HbF levels is not yet fully understood. Low MYB expression has been detected in the erythroid cells of individuals with high HbF levels [74,75]. An analysis of c-MYB loss-of-function studies [66,73,75] shows that some of the c-MYB-related γ-globin inhibitory genes, i.e., BCL11A are downregulated upon MYB depletion. Hence, c-MYB directly activates key γ-globin repressor genes. Furthermore, the knockdown of the MYB gene in human erythroid progenitors causes elevated HbF [76]. It is considered a major modifier of the severity of β-hemoglobinopathies, due to its role in regulating HbF gene expression [77].

The epigenetic modifiers of hemoglobin expression involve the DNA methylation process, Lysine-specific demethylase (LSD1), and histone deacetylases 1/2 (HDACs1/2) [78]. Through the DNA methyltransferases (DNMT3A, DNMT3B, DNMT1, and MBD family), a methyl group is transferred to the promoter of γ-globin genes, leading to the epigenetic silencing of HbF. DNA methylation inhibitors restore the normal amounts of HbF [79]. HDACs1/2 act in the same way, thereby repressing γ-globin expression. Consequently, the chemical inhibition of HDACs1/2 induces HBF and γ-globin expression, suggesting that this action is regulated by the histone acetylation-induced triggering of the GATA2 gene [80]. Finally, Lysine-specific demethylase (LSD1), after its interaction with HDACs1/2, BCL11A, and the epigenetic DRED complex, inhibits HbF and γ-globin expression. Thus, LSD1 inhibition by tranylcypromine in human erythroid cells or in transgenic mice increases γ-globin expression and HbF. LSD1 is an important therapeutic element for γ-globin production, and it may serve as a new γ-globin and HbF inducer in patients with hemoglobinopathies [81].

LIM Domain Binding-1 (LDB1) is found on chromosome 10q24.32, and it is a co-factor that interacts with the erythroid activators KLF1, GATA-1, and FOG-1 in order to determine the proximity between the LCR and the β-globin gene [118,119,120]. The latter is a prerequisite for nuclear migration and transcription triggering of the β-globin gene [121,122]. This chromosomal looping protein (LDB1) is necessary for β-globin gene activation during erythroid differentiation [123,124] through mechanisms suggesting the establishment of the proximity between the LCR and globin genes and relocation of the locus inside the nucleus [121]. A critical component of globin switching is the LCR enhancer’s contact with γ-globin and β-globin genes [20,125]. Deng et al. worked with a zinc-finger DNA-binding peptide (γZnF) to direct an LDB1 dimer to the silenced γ-globin gene promoters in adult erythroid progenitors, and the γ-globin gene was reactivated [126]. This is important because continuous HbF production in adult life, as it occurs in HPFH, improves the clinical course of β-hemoglobinopathies [127].

Butyrates were first identified as chemical histone deacetylase inhibitors (HDACis) [82], accompanied by the modification of the chromatin structure and transcriptional events [83]. Butyrates have been examined on K562 cells and on erythroid progenitor cells [84,85]. The ability of sodium butyrate and α-amino-n-butyric acid (ABA) to increase γ-globin synthesis in vitro in the erythroid progenitors of β-thalassemic and SCD patients established the therapeutic potential of these molecules [86]. Several studies defined the doses able to induce HbF production with less cytotoxic side effects. In addition, the efficacy and toxicity of butyric acid were evaluated [87]. Various in vivo experiments using mouse [88], primate, and human models [89,90,91] demonstrated the expected clinical effects of this class of HbF inducers. Later studies showed that one out of four patients did not respond after being treated with butyrates [92]. A more recent study observed that patients who do not respond to hydroxyurea (HU) treatment can be treated with sodium butyrate containing microRNAs to increase HbF synthesis [93]. Apart from in vitro and in vivo models, butyrates, displaying an analogous mechanism of action, have been tested in clinical trials [94,95,96,97,98]. Oral isobutyramide has been used to treat patients with TI in order to increase the degree of HbF induction [99]. Interestingly, the loss-of-function variants in SUPT5H serve as modifying factors in β-thalassemia. Patients with combined heterozygosity of SUPT5H and HBB gene variants demonstrate a mild β-TI phenotype [100].

The Widely Interspaced Zinc Finger-Containing Protein (WIZ) is a transcription factor found on the chromosome 19p13.12, close to the EKLF/KLF1 and the ZBTB7A transcription factors. The WIZ was discovered as a repressor of HbF by the phenotypic screening of a Cereblon-biased chemical library and target deconvolution by global proteomics. The degradation of the WIZ is a promising therapeutic strategy for SCD patients. dWIZ-1 and dWIZ-2 are molecular glue degraders of the WIZ transcription factor and robust HbF inducers with the following mechanisms [101]. WIZ degradation is controlled by the recruitment of WIZ to CRBN by dWIZ-1. Furthermore, the WIZ binds to the β-globin locus and favors repressive chromatin methylation. The pharmacological inhibition of the WIZ has no significant side effects and induces HbF in cynomolgus monkeys and humanized mice [101].

MicroRNAs (miRNAs) are intracellular non-coding RNAs playing an important role in gene expression by targeting specific mRNA regions [102], demonstrating a translational inhibition of mRNA [26]. They are associated with erythroid differentiation [103]. They also play a role in HbF regulation [104] and in the pathophysiology of several β-hemoglobinopathies. For instance, miR-451 is upregulated in thalassemic erythroid progenitor cells, in comparison to normal cells [105], whereas the expressed in β-thalassemia miR-210 is upregulated in hypoxic conditions [106]. Moreover, they are implicated in the epigenetic mechanisms of HGB gene activation [107]. miRNA expression profile analysis identified 12 differentially expressed miRNAs in HbE/β-thalassemia with high HbF levels, compared to those with normal HbF levels, which might be involved in the regulation of HbF expression [108]. There is an extensive scientific debate on how miRNAs control γ-globin expression in β-thalassemic patients [104]. miRNA therapy for β-thalassemia targets several regulatory genes, such as BCL11A, GATA1, and SOX6 [109]. For example, miR-210-3p [110], miR-92a-3p [111], and miR-30a [112] target BCL11A mRNA and, in this way, γ-globin expression is upregulated. These miRNAs are promising molecules for therapeutic intervention and potent pharmacological targets to stimulate HbF production.

α-globin gene expression and modifiers: α-globin gene expression and α-globin modifiers have a strong impact on the phenotype and clinical severity of anemia in patients with β-thalassemia or SCD. Thus, the inheritance of additional alpha genes, like in α-gene triplication, causes an excess of free α-globin chains, which is responsible for more severe hemolysis, anemia, and ineffective erythropoiesis, forming the hallmark of the disease. On the contrary, the inheritance of alpha globin gene variants, causing the reduction in or absence of alpha globin synthesis, has been linked to milder clinical manifestations in beta thalassemic or SCD patients [113,114]. The decrease in the toxic excess of free α-globins is demonstrated in β-thalassemic cells by the activation of autophagy or by an increased expression of the α-globin-stabilizing protein (AHSP) gene, which is an α-globin genetic modifier and a strong candidate for targeted therapy. Interestingly, unc-51-like autophagy activating kinase 1 (ULK1) is necessary for the induction of autophagy in erythroid cells [113]. Furthermore, the loss of miR-144/451 stimulates the ULK1-mediated autophagy of free α-globins and ameliorates the symptoms of β-thalassemia [115]. A detailed description of the α-globin genetic modifiers in relation to α-globin gene expression has been extensively published in the past [113] and is beyond the scope of this review.

## 4. Therapeutic Interventions

Although the underlying molecular basis of both SCD and thalassemia has been associated with disease presentation, epigenetic factors might affect disease manifestations, creating different clinical pictures, in spite of the presence of similar underlying mutations [128]. Epigenetic regulation acts via several mechanisms, which cause chromatin modifications and either allow or prevent transcription [129]. DNA methylation (CPG islands) and histone modification (acetylated or methylated lysine residues in N-terminal tails) result in the modification of gene triggering without an alteration in the DNA sequence [130]. Specifically, CpG methylation suppresses genes by preventing the binding of transcription factors. On the contrary, CpGs in actively transcribed gene promoter regions are mainly unmethylated. Moreover, acetylated lysine residues result in active gene transcription, and methylated ones can be associated with active or repressed gene transcription [131].

Several clinical studies demonstrate that HbF is a modulator in the clinical course of haemoglobinopathy. Regarding SCD, the enhanced synthesis of HbF blocks the polymerization process and reduces red cell sickling, thereby ameliorating the symptoms of the disease. In addition, increased levels of γ-globin expression, resulting in an elevated synthesis of HbF in β-thalassemia, decrease α- and β-chain imbalances and improve the clinical phenotype. Epigenetic modifiers (DNA hypomethylation and histone acetylation) are efficient in causing γ-globin expression to reproduce HbF [132,133,134]. The relevant drugs and the mechanisms of genetic modifications affecting hemoglobin expression are described below.

### 4.1. Hydroxyurea (HU)

HU is the most broadly used small molecule, inducing the expression of HbF for more than 30 years. Although the precise molecular mechanisms underlining the induction of HbF remain unclear, the inhibition of BCL11A and MAP3K5 could be involved [134,135]. HU is currently the only European Medicines Agency (EMA) and U.S. Food and Drug Administration (FDA)-approved HbF-inducing drug for individuals with SCD [136]. Patients treated with HU show a remarkable decrease in the prevalence of acute painful crises, a relief of symptoms, and the prevention of organ damage. In addition, they have reduced mortality after long-term follow-up treatment [137,138]. However, HU, with its variable efficacy, causes dose-limiting myelosuppression [50,139]. Furthermore, the efficacy of HU in the treatment of β-thalassemic patients is low because of the drug’s incapability to demonstrate α- and β-chain balances with low HbF production. Hence, the development of more targeted and efficacious HbF inducers is necessary.

### 4.2. DNA Methylation

Fetal hemoglobin is epigenetically silenced by DNA methyltransferase (DNMT) in adult erythropoiesis. DNMT inhibitors 5-azacytidine (5-aza) and 5-aza-2′-deoxycytidine (decitabine) produce elevated HbF expression in preclinical models and clinical studies. Nevertheless, they are not approved at present for the treatment of β-thalassemia and SCD. Decitabine was well-tolerated and effective in elevating HbF levels in β-thalassemia [140] and SCD [141] clinical trials. However, decitabine increased the platelet count, and it is not approved due to concerns regarding its adverse effects. GSK3482364 is a novel. orally bioavailable DNMT1-selective inhibitor currently being tested in pre-clinical studies [142].

Decitabine is quickly metabolized in vivo by the enzyme cytidine deaminase (CDA), which severely limits its oral bioavailability, compared with intravenous administration. Co-administration of the CDA inhibitor tetrahydrouridine (THU) enhances decitabine bioavailability. Clinical trials have shown that the oral administration of combined decitabine and THU is suitable for DNMT1-targeted therapy aiming at increasing HbF in SCD patients [143]. A multicentric phase II trial evaluating the efficacy and safety of oral decitabine and tetrahydrouridine (NDec) in patients with SCD (ASCENT1) is underway (NCT05405114, clinicaltrials.gov).

### 4.3. Histone Modification

Vorinostat, a pan-histone deacetylase inhibitor (HDACi), has been used in clinical trials aiming at reactivating HbF expression. Nevertheless, the relevant clinical trial on SCD patients was terminated because of the recruitment of a very small number of patients [144]. Interestingly, CT-101, a novel class I-restricted HDACi, which is a Largazole derivative, produced HbF with additive activity combined with HU in preclinical studies (SCD-derived erythroid progenitors). CT-101 might exhibit advantages in the clinical setting, compared to other pan-HDACis, because of its efficacy at nanomolar concentrations and its selectivity profile, compared to first-generation HDACis [145].

Sodium butyrate, another HDACi, has been investigated as a potential treatment for SCD, due to its capability of inducing HbF. Butyrate increases by three-fold the levels of HbF and the proportion of HbF among total hemoglobin. Furthermore, it demonstrates reduced hospitalization rates, establishing its therapeutic advantages [146]. Nevertheless, the need for intravenous administration limits its future expansion. MS-275 is another HDACi, inducing HbF production. Its analog MD-48 shows the highest activity because it statistically increases hemoglobin production by almost up to 14 times comparatively [147].

Furthermore, the IOX1 histone demethylase inhibitor down-regulates α- and α-like globin expressions, exclusive of influencing the β-like globin expression or affecting erythroid differentiation. These data hint that IOX1 target might serve as an important element for the treatment of β-thalassemia [148].

A novel protein deacetylase, namely SIRT1, increases γ-globin expression when it is over-expressed. Preclinical studies using SIRT1 activator molecules, like SRT2104 or SRT1720, have shown the reactivation of the γ-globin gene. These molecules have great potential as new HbF inducers. SRT1 forms LCR loops targeting the γ-globin gene promoter and triggering the gene. Additionally, simultaneously, the expression of γ-globin gene inhibitors (BCL11A, KLF1, HDAC1, and HDAC2) is suppressed, and the latter results in the increase in histone acetylation on the γ-globin promoter [149].

Other drugs, modulating histone methylations, have also been used to trigger the expression of HbF in preclinical studies using animals, and can be developed further, like RN-1, a potent irreversible inhibitor of LSD1 [150,151,152], or FTX-6058, an inhibitor of EED [153] and UNC0638, a selective suppressor of EHMT1/2 histone methyltransferases [154,155]. Furthermore, UNC0638 leads to increased HbF expression in erythroid cells derived from β-thalassemic patients [156]. Nevertheless, the mode of action of these inhibitors is often vague, due to a lack of specificity. Moreover, their pleiotropic role in cell fitness poses serious safety concerns [157]. Epigenome editing is more targeted, compared to drugs.

### 4.4. Thalidomide and Other Immunomodulatory Drugs (IMiDs)

Thalidomide is a potent agent for enhancing the proliferation of erythroid precursors and HbF upregulation by markedly decreasing the H3K27 methylation of γ-globin gene promoters [158,159]. Moreover, pomalidomide and lenalidomide control erythropoiesis and HbF production in human CD34 cells. Hence, they can improve β-globin imbalance in hemoglobinopathies [160].

### 4.5. Other Pharmacological Agents

#### 4.5.1. Cilostazol

Cilostazol (CTZ, OPC-13013), a quinolinone derivative [161], is a reversible type III phosphodiesterase suppressor. It acts as an antiplatelet agent with vasodilating properties, and it is an approved drug for treating chronic arterial occlusion [162]. The antiplatelet and vasodilatory capacities of CTZ, along with HbF induction, as well as the oral availability and the relatively low toxicity [163], suggest that CTZ may represent a promising HbF inducer to be developed for β-thalassemic and SCD patients.

#### 4.5.2. Sirolimus

Sirolimus is a mammalian target of the rapamycin (mTOR) inhibitor. Preclinical and early clinical studies have showed that it elevates HbF in cultures from β-thalassemic patients with various HbF levels; sirolimus elevates the overall hemoglobin content per cell and might increase the clearance of α-globin in red blood cell precursors, with only minor effects on β-globin, resulting in the mitigation of ineffective erythropoiesis in thalassemic patients [164,165,166]. In this regard, two phase II trials of sirolimus on patients with transfusion-dependent thalassemia (TDT) (NCT03877809, NCT04247750) are underway. They will investigate possible HbF induction, along with the ineffective hematopoiesis attributed to the drug [164].

#### 4.5.3. IMR-687 and Benserazide

Another target for HbF production might be phosphodiesterase 9’s inhibition with IMR-687. A phase II trial of IMR-687 using patients with TDT and non-transfusion-dependent thalassemia (NTDT) is currently underway (NCT04411082) [164,167,168]. In addition, benserazide, which is currently used in combination with levo-dopa for the treatment of Parkinson’s disease, demonstrated a good induction of HbF. A phase I trial of benserazide using patients with NTDT is ongoing (NCT04432623) [164,169,170].

**Figure 4:**
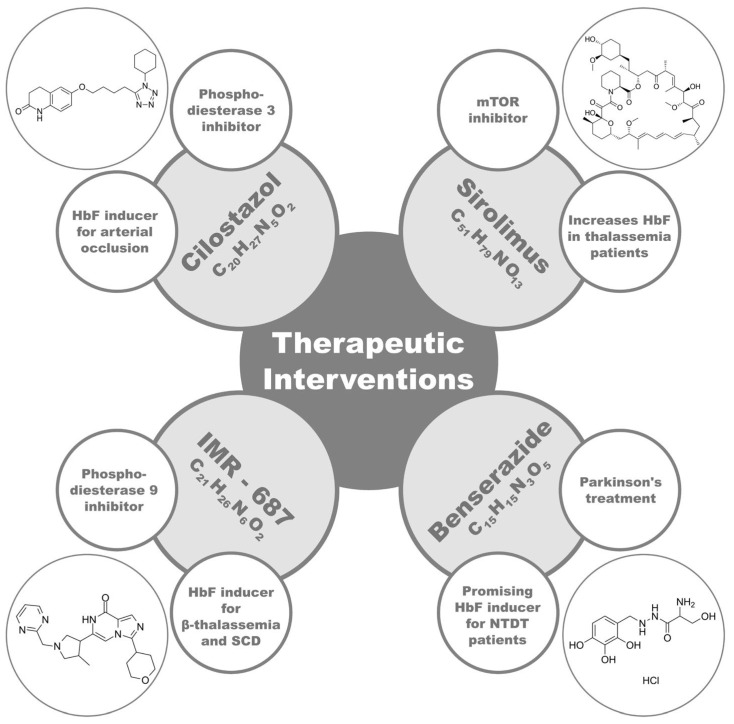
The most important elements of CTZ, Sirolimus, IMR-687, and benserazide.

Figure 4 provides the most important elements of CTZ, Sirolimus, IMR-687, and benserazide.

## 5. Gene Therapy—Gene Editing

### 5.1. Gene Therapy

Gene therapy aims for the improvement of the synthesis of various globin types or targets defective β-globin genes. The expression of globin genes and the clinical manifestations of the disease are regulated by genetic modifiers, which are essential for the efficacy of gene therapy for patients with β-thalassemia and SCD [171,172]. The most common method involves the insertion of a functional copy of the β-globin gene into the patient’s hematopoietic stem cells (HSCs) via lentiviral vectors [173]. The procedure consists of several phases, as follows.

The first step is HSC collection. The patient’s HSCs are collected through either bone marrow aspiration or mobilized peripheral blood collection. The mobilization of peripheral blood is accomplished by administering growth factors, such as the granulocyte colony-stimulating factor (G-CSF), which promote the release of HSCs into the circulation [174,175].

Gene transfer is the following phase. Following the harvesting process, the HSCs undergo genetic modification with lentiviral vectors, which transport the β-globin gene, disrupting HbF switching or fixing sickle hemoglobin mutation. These vectors, originating from the human immunodeficiency virus-1 (HIV-1), are engineered to integrate into the host genome, enabling the stable and long-term expression of the newly inserted gene. This ascertains that the production of β-globin is at a sufficient level for treating thalassemia symptoms in the case of thalassemic patients [175,176].

Upon the completion of the gene modification procedure, the genetically corrected HSCs are reintroduced into the patient via intravenous infusion, after the patient has undergone myeloablative conditioning. In other words, gene therapy is combined with autologous hematopoietic stem cell transplantation (HSCT). These cells eventually engraft into the bone marrow. Erythrocytes capable of synthesizing functional β-globin chains are the evolution of the primary, genetically modified, inserted cells. This method has shown significant effectiveness in clinical trials, with certain patients attaining transfusion independence and elevated hemoglobin levels [172,177,178,179,180]. Because the patients serve as their own donors, there is no need for immunosuppression or any risk of graft versus host disease (GvHD) [177].

### 5.2. Gene Editing

Gene editing offers a more precise method for correcting genetic defects linked to thalassemia or SCD. The emergence of innovative technology, such as CRISPR/Cas9 and base editing, has enabled a long-lasting modification of the genome, providing the possibility for the permanent repair of molecular defects [174]. The primary gene editing methodologies can be categorized into the following classifications.

The CRISPR/Cas9 method uses guide RNA that directs the Cas9 nuclease to a specific genomic location, resulting in a double-strand DNA break. The cell’s intrinsic repair mechanisms are used to either insert a repaired DNA sequence via homology-directed repair (HDR) or to deactivate the defective gene. CRISPR/Cas9 can directly target the β-globin gene or the α-globin gene to restore the balance between α- and β-globin chains and normalize hemoglobin production in thalassemic syndromes. This method can also be applied in SCD [173,176,181].

The latest development in this technology is base editing, which enables the accurate transformation of one DNA base pair into another with no double-strand breaks. This technique uses modified deaminases to allow precise gene modifications, including the rectification of point mutations in the β-globin gene. One of the primary advantages associated with base editing is its reduced possibility of off-target effects, significantly improving its safety for clinical use [182,183,184].

Moreover, the reactivation of the γ-globin gene, which codes for HbF, is another possible gene editing approach [153,185]. An increase in HbF levels can be used to enhance clinical outcomes in situations where β-globin is lacking. Using gene editing techniques, it is possible to elevate HbF levels and reduce the symptoms of β-thalassemia or SCD by targeting the transcriptional repressor BCL11A [104,173,174,186].

Recently, researchers have examined the potential of combination therapies, which are intended to reduce the production of α-globin and introduce functional β-globin within the same treatment regimen. This dual strategy might reduce the essential β-globin threshold for effective therapy, providing an advanced form of genome editing to target numerous pathways involved in hemoglobinopathies [104,176,185].

Table 2 shows the gene addition or gene editing therapeutic strategies for hemoglobinopathies and describes the mechanism of action and the relevant technology and cost, providing information of the date of FDA and EMA approval [171,172,180,186].

## 6. Conclusions

In this review, we focused on the genetic modifiers of hemoglobin expression from a clinical perspective, and provided an overview of the association between the complex molecular mechanisms of these molecules in relation to targeted drug therapy in order to induce normal levels of hemoglobin in patients with hemoglobinopathies. This work was written on behalf of the International Hemoglobinopathy Research Network (INHERENT) project, aiming at deciphering the role of genetic modifiers in hemoglobinopathies through a large, global, genome-wide association study (GWAS) [187]. By acquiring a better understanding of the molecular defects of hemoglobinopathies and the complex network the genetic modifiers of hemoglobin compose during their normal or abnormal functions, the discovery of novel targeted drugs, aiming at ameliorating the lives of patients with hemoglobinopathies, can be achieved.

## Figures and Tables

**Table 2:** Gene addition and gene editing therapeutic strategies for hemoglobinopathies.

Gene Editing/ Gene Addition	Disease	Mechanism of Action	Cost	NCT Code—References	Approval
ΒΕΤΙ-CEL	TDT β-thalassemia Non-β0/β0	Lentiviral-based gene addition	USD 2.8 MILLION	NCT02906202 Phase III—(ref. [172])	FDA (August 2022) EMA (approval 2019, cancellation 2022)
LOVO-CEL	SCD	Lentiviral-based gene addition (HbAT87Q)	USD 3.1 MILLION	NCT04293185 Phases I/II (ref. [171])	FDA (December 2023) EMA (-)
EXA-CEL	TDT β-thalassemia SCD	CrispR/Cas9 gene editing (BCL11A—HbF axis)	USD 2.2 MILLION	NCT05951205 Phases I/II (refs. [180,186])	FDA (December 2023, January 2024) EMA (2024)

## Data Availability

Not applicable.

## Abbreviations

5-AZA	5-Azacytidine
ABA	α-Amino-N-Butyric Acid
AHSP	α-Globin-Stabilizing Protein
BCL11A	B Cell Lymphoma/Leukemia 11A
C-MYB	Cellular Myelocytomatosis Oncogene
C2H2-TYPE ZINC FINGER PROTEIN	Acetylene-Type Zinc Finger Protein
CDA	Cytidine Deaminase
COREST	Corepressor Complex
CPG	Carry Propagate and Generate Processor Unit
CRB1	Crumbs Homolog 1
CRISPR/CAS9	CRISPR-Associated Protein 9
CTZ	Cilostazol
DECITABINE	5-Aza-2′-Deoxycytidine
DRED	Direct Repeat Erythroid Definitive
DNMT1	Deoxynucleic Acid Methyltransferase 1
DNMT3A	DNA Methyltransferase 3 Alpha
DNMT3B	DNA Methyltransferase 3 Beta
E2A	E2A Immunoglobulin Enhancer-Binding Factors E12/E47
EED	Embryonic Ectoderm Development
EHMTs1/2	Histone Methyltransferases
EMA	European Medicines Agency
EKFL	Erythroid Krueppel-Like Factor 1
FDA	U.S. Food and Drug Administration
FOG = ZFPFM1	Zinc Finger Protein Family Member 1
G-CSF	Granulocyte Colony-Stimulating Factor
GATA1	GATA Binding Factor 1 or Erythroid Transcription Factor
GSK3482364	The Purified R-Enantiomer of GSK-3482364
GVHD	Graft Versus Host Disease
HBA	Adult Hemoglobin
HBA2	Hemoglobin A2
HBF	Fetal Hemoglobin
HBS1L-MYB	HBS1 Like Translational GTPase-Myeloblastosis
HBG1	Hemoglobin Subunit Gamma 1
HBG2	Hemoglobin Subunit Gamma 2
HDAC1	Histone Deacetylase 1
HDAC2	Histone Deacetylase 2
HDACS1/2	The Histone Deacetylases 1/2
HDACI	Pan-Histone Deacetylase Inhibitor
HDR	Homology-Directed Repair
HIC2	Hypermethylated in Cancer 2
HIRA	Histone Chaperone Complex Cell Cycle Regulator
HIV-1	Human Immunodeficiency Virus-1
HPFH	Hereditary Persistence of Fetal Hemoglobin
HSCs	Hematopoietic Stem Cells
HSCT	Hematopoietic Stem Cell Transplantation
HU	Hydroxyurea
HUF	Hereditary Unresponsiveness of Fetal Hemoglobin
IOX1	Histone Demethylase Inhibitor
K562 CELLS	Erythroleukemia Cell Line That Lacks HLA Antigen Expression
KLF1	Krueppel-Like Factor 1
LCR	Locus Control Region
LDB1	LIM Domain Binding 1
LMO2	LIM Domain Only 2
LRF	Leukemia/Lymphoma-Related Factor
LSD1	Lysine-Specific Demethylase 1A
MAP3K5	Mitogen-Activated Protein Kinase 5
MBD FAMILY	Methyl-CpG-Binding Domain Protein
MIR-144/451	Bona Fide Tumor-Suppressing Gene
MIRNA	Micro RNA
MTOR	Target of Rapamycin
NF-E2	Nuclear-Factor Erythroid 2 Transcription Factor
NDEC	Tetrahydrouridine
NURD	Nucleosome Remodeling and Deacetylation Complex
NTDT	Non-Transfusion-Dependent Thalassemia
PK	Pyruvate Kinase
SCD	Sickle Cell Disease
SCL	Stem Cell Leukemia
SIRT1	Silent Information Regulator Sirtuin 1 Protein
SNPs	Single Nucleotide Polymorphisms
SOX	SRY-Related High-Mobility Group (HMG) Box
SOX6	SRY-Box Transcription Factor 6
SUPT5H	SPT5 Homolog, DSIF Elongation Factor Subunit
TAL1	T-Cell Acute Lymphocytic Leukemia Protein
TDT	Transfusion-Dependent Thalassemia
THU	Tetrahydrouridine
TR2/TR4	Testicular Nuclear Receptors 2/4
ULK1	Like Autophagy Activating Kinase 1
WIZ	Widely Interspaced Zinc Finger-Containing Protein
ZBTB7A	Transcription Factor Zinc Finger and BTB Domain Containing 7A
